# Effect of medication adherence on quality of life, activation measures, and health imagine in the elderly people: a cross-sectional study

**DOI:** 10.1186/s12877-024-05227-3

**Published:** 2024-07-24

**Authors:** Muayad Saud Albadrani, Yousef Omar Aljeelani, Safwan Hatem Farsi, Mohammed Ali Aljohani, Abdulrahman Abdullah Qarh, Ahmed Saleh Aljohani, Abdulrahman Awadallah Alharbi, Muhammad Abubaker A. Tobaiqi, Atallah Mohammad Aljohani, Naweed SyedKhaleel Alzaman, Hammad Ali Fadlalmola

**Affiliations:** 1 Department of Family and Community Medicine and Medical Education College of Medicine, Taibah University, Madinah, Saudi Arabia; 2 Preventive Medicine Clinics Complex Madinah Health Cluster, Madinah, Saudi Arabia; 3 College of Medicine, Taibah University, Madinah, Saudi Arabia; 4 Department of Internal Medicine, College of Medicine, Taibah University, Madinah, Saudi Arabia; 5 Nursing college, Taibah University, Madinah, Saudi Arabia

**Keywords:** MMAS-8, PAM13, EQ-5D-5L, Medication adherence, Cross-sectional

## Abstract

**Background:**

Usually, old age brings a poor quality of life due to illness and frailty. To prolong their lives and ensure their survival, all elderly patients with chronic diseases must adhere to their medications. In our study, we investigate medication adherence for elderly patients and its impact on the general health of the patient.

**Methods:**

We implemented a cross-sectional survey‐based study with four sections in April 2022 in Saudi Arabia. Data about the participants’ demographic characteristics, the Morisky Medication Adherence Scale, Patient Activation Measure (PAM) 13, and EQ-5D-5 L.

**Results:**

A total of 421 patients participated in this study, their mean age was 60.4 years, and most of them were males. Most of our population is living independently 87.9%. The vast majority of people have a low adherence record in the Morisky Medication Adherence Scale (8-MMAS) classes (score = < 6). Moreover, the average PAM13 score is 51.93 (Level2) indicating a low level of confidence and sufficient knowledge to take action. Our analysis showed a significant correlation between socioeconomic status and medication adherence. Also, there was an association between housing status and medication adherence. On the other hand, we found no correlation between medication adherence and quality of life (QOL) by EQ-5D-5 L.

**Conclusion:**

Medication adherence is directly affected by living arrangements, as patients who live with a caretaker who can remind them to take their medications at the appropriate times have better medication adherence than those who live alone. Medication adherence was also significantly influenced by socioeconomic status, perhaps as a result of psychological effects and the belief of the lower-salaried population that they would be unable to afford the additional money required to cure any comorbidities that arose as a result of the disease. On the other hand, we did not find any correlation between medication adherence and quality of life. Finally, awareness of the necessity of adherence to medication for the elderly is essential.

## Introduction

Aging is a natural process that begins in early adulthood and continues throughout the rest of life. Many biological functions begin to gradually decline in early middle age. There is no set age at which a person is considered elderly [1, 2]. Traditionally, old age is considered to begin at 65; when all different body functions start to decline, and when humans are more susceptible to the occurrence of death [3]. Age is one of the most significant risk factors for cardiovascular disease (CVD). By 2030, approximately one-fifth of the global population will be 65 or older, and the prevalence of CVD will increase exponentially [4]. Heart disease, stroke, cancer, and diabetes are among the most frequent and expensive chronic diseases affecting the elderly, and 92% of them have at least one, according to the National Council on Aging. The number of old aged people is increasing yearly, due to the increase in medical attention toward the extension of life. It is expected that the number of old aged people will pass the number of children by the year 2050 [5].

Illness and frailty often reduce the quality of life in old age. Many elderly people have chronic diseases that require them to take medication every day, and some need to do it more than once [6]. All old aged patients with chronic disease require medication adherence to extend their life and survivability. Although medication adherence is important, nearly 50% of the patients receiving medication are non-adherent [7]. In most cases, medication adherence decreases due to the memory of the patient and forgetfulness. On the other hand, nonadherence can be an intentional choice made by the patient [5]. Medication adherence in elders can be even less due to the reduction in income after retirement and a decrease in food intake for financial reasons [8], in addition to the poor knowledge about the risk factors and the complications that may result if they stopped taking medication on their own [9]. Quality of life has emerged as a critical outcome metric for evaluating the success of illness management strategies as patients with chronic diseases are living longer and receiving treatment for longer periods [6, 10].

A previous study that evaluated medication adherence in Lebanese patients showed that 22.4% of the population with low adherence to the medication [11].

Medication adherence varies widely across different countries and regions, depending on various factors such as the health care system, the patient’s characteristics, the medication characteristics, and the social and economic environment. For example, the rate of medication adherence in the United States in elderly patients was 88.2–90.4% [12, 13], In Italy was 60% [14], In Uganda was 18.2% [15], In Palestine was 48% [16], In a previous study in Saudi Arabia was 57% [17]. We noticed that the majority of elderly people in Saudi Arabia had low adherence to medication, which considers a major challenge in the treatment of older patients.

Therefore, our study differs from the previous ones and will contribute to the scientific literature by using a validated instrument to measure medication adherence among elderly patients with chronic diseases in Saudi Arabia, and by comparing our findings with global and regional trends. Our study also aims to provide a comprehensive assessment of the factors associated with medication adherence, such as socio-demographic characteristics, health status, medication-related factors, and health beliefs.

Several factors can influence adherence such as socioeconomic status. Moreover, medication adherence can also increase patient activation by enhancing knowledge, skills, and confidence in disease self-management. We aim to assess medication adherence in elderly people and the percentage of low, moderate, and high-adherent patients as well as the medication adherence impact on the quality of life and activation measures.

## Methods

### Study design and setting

A self-administered, piloted, and completely anonymous questionnaire was used to conduct a cross-sectional study. Following the STROBE guidelines for reporting and conducting cross-sectional studies [18], we conducted this study. Patients at a Saudi Arabian hospital and members of the general public participated in the online survey.

### Inclusion and exclusion criteria

We included patients who are diagnosed either with diabetes mellitus, or cardiovascular diseases such as hypertension, atherosclerosis, and cerebrovascular disease. Also, the age should be more than 50 years old. On the other hand, we excluded the following:

Patients who were in the hospital and received medications from healthcare workers instead of taking them by themselves, or patients who had mental disorders, hearing impairments, were older than 80 years, or had someone else administer their medications regularly.

### Study instruments

The used questionnaire was formed of four sections as follows:

1. Demographic characteristics include age, sex, nationality, employment, socioeconomic status, education, and housing status.
2. The Morisky Medication Adherence Scale (MMAS) is a highly valid and reliable assessment tool aimed to screen medication adherence in a variety of populations [19–21]. The tool uses some short questions geared in a way to avoid “yes-saying” bias which is a barrier in chronic disease patient care. Patients with perfect adherence received an 8, while the lowest possible adherence was given a score of 0. With each decrease in scores, patient adherence to their medical treatment decreased. The users’ adherence to the MMAS-8 guidelines was categorized from high (8 points) to medium (7 or 6 points) to low (5 points or below). We used the Arabic version of Morisky Medication Adherence Scale (MMAS) which was shown to be valid and reliable in Arabic populations [22].
3. The Patient Activation Measure (PAM) is a valid, highly reliable, unidimensional, probabilistic Guttman-like scale that reflects a developmental model of activation. The PAM13 shows good reliability and validity for measuring patient activation in patients with hypertension and/or diabetes. The PAM score’s typical intervals were used to assign value to each of the final four categories. Those with a level 1 score (between 0 and 47) demonstrate a minimal appreciation for the value of patient level. Level 2 (47.1–55.1) indicates a lack of self-level and insufficient expertise to proceed. When a patient reaches level 3 (55.2–72.4), they show signs of positive behavioral change and are beginning to implement healthcare recommendations. A level 4 (72.5–100) indicates that the patient taking charge of his/her health [23, 24].
4. The EQ-5D-5 L was used as a generic tool for describing and valuing health in terms of five dimensions: mobility, self-care, usual activities, pain/discomfort, and anxiety/depression and each dimension had five levels; no problem, slight problems, moderate problems, severe problems, and extreme problems/unable to do [25]. We used the Arabic version of the EQ-5D-5 L tool which was shown to be valid and reliable in Saudi Arabia populations [26].

### Sampling and sample size calculation

Based on a population size of 200,000, a Confidence level of 95%, an anticipated frequency of 50%, a confidence limit of 5%, and a design effect of 1, the sample size of 384 was considered the minimum required sample.

### Data collection and handling

The study took place in Saudi Arabia, Al Madinah. The duration was less than one year starting April 2022. The questionnaire was distributed to the infirmary patients and community through online electronic forms. We distributed the form through social media groups, especially those known to elderly people. Also, we clarified that only elderly people should fill out this form. After collecting all responses, we filtered out them according to age.

### Statistical analysis

After cleaning the data, the statistical analysis was performed using Statistical Package for Social Sciences software (IBM SPSS Statistics for Windows, Armonk, New York, US), version 26. Continuous variables were expressed as mean and standard deviation, whereas categorical variables were expressed as frequency and percent. Descriptive analyses were conducted (frequency and percentage) to describe demographic characteristics. A Chi-square test was used to explore the relationship between medication adherence and demographic characteristics and quality of life (QOL) by EQ-5D-5 L. The *P*-value was considered statistically significant if less than 0.05.

## Results

### Patients characteristics

A total of 421 patients participated in this study, most of them from Saudi Arabia, the mean age of the population was 60.4 years (SD = 8.1), and most of the study population was males (69.8%). Only four patients were non-Saudi. Nearly half of the population is retired (53.9%) and some of them are still working (26.8%). The economic status of our population is variable with a majority of the population with income of fewer than 5000 Saudi riyals (SR) per month (31.1%) and the smallest number in our population were the people with more than 20,000 SR per month (9.5%). Our entire population had education grades and the majority of the population had college grades (49.2%). Most of our population is living independently (87.9%) which may inflict on the medication adherence of these patients with no one to remind them for taking their medication. Table 1.

**Table 1:** Patients characteristics

Basic characteristics	Mean ± SD, or *N* (%)
**Age** (year)	60.4 ± 8.1
**Sex**	
• Female	127 (30.2)
• Male	294 (69.8)
**Nationality**	
• Saudi	417 (99)
• Non-Saudi	4 (1)
**Employment**	
• Unemployed	81 (19.2)
• Employed	113 (26.8)
• Retired	227 (53.9)
**Socioeconomic status**	
• Less than 5000	131 (31.1)
• 5000- <10,000	105 (24.9)
• 10,000- <15,000	88 (20.9)
• 15,000–20,000	57 (13.5)
• More than 20,000	40 (9.5)
**Education**	
• None	0
• Primary school	50 (11.9)
• Secondary school	49 (11.6)
• High school	115 (27.3)
• College	207 (49.2)
**Housing status**	
• Independent	370 (87.9)
• Under constant care	46 (10.9)
• Elderly care home	5 (1.2)

### Percentages of 8-MMAS answers and adherence scores

In the medical adherence Sect. (8-MMAS) most of our population was found to forget to take their medication with a percentage (72.4%). They not only forget sometimes, but also they miss the medication due to other reasons (64.1%). Sometimes they stop taking medication; because they feel that medication makes their condition even worse, and without the knowledge of the doctor that follows up on their medical condition. On the other hand, a high percentage stop taking their medication due to opposite reasons as they feel they are better or their medical condition is under control (65.3%). From all these results with a majority of the patients forgetting their medication or not sticking to their medical plans, most of our population were of low adherence (score = < 6) with a percentage of (84.8%) in the 8-MMAS classes. Table 2.

**Table 2:** Percentages of 8-MMAS answers and adherence scores

Percentages of 8-MMAS answers and adherence scores	*N* (%)
Do you sometimes forget to take your medications?	
• **Yes**	305 (72.4)
• **No**	116 (27.6)
**People sometimes miss taking their medications for reasons other than forgetting. Thinking over the past two weeks**,** were there any days when you did not take your medications?**	
• **Yes**	270 (64.1)
• **No**	151 (35.9)
**Have you ever cut back or stopped taking your medications without telling your doctor**,** because you felt worse when you took it?**	
• **Yes**	281 (66.7)
• **No**	140 (33.3)
**When you travel or leave home**,** do you sometimes forget to bring along your medications?**	
• **Yes**	292 (69.4)
• **No**	129 (30.6)
**Did you take your medications yesterday?**	
• **Yes**	378 (89.8)
• **No**	43 (10.2)
**When you feel like your health condition is under control**,** do you sometimes stop taking your medications?**	
• **Yes**	275 (65.3)
• **No**	146 (34.7)
**Taking medications every day is a real inconvenience for some people. Do you ever feel hassled about sticking to your treatment plan?**	
• **Yes**	318 (75.5)
• **No**	103 (24.5)
**Do you have difficulty remembering to take all your medications?**	
• **Yes**	267 (63.4)
• **No**	154 (36.6)
**8-MMAS classes (Medication adherence level)**	
• **Low adherence (score = < 6)**	357 (84.8)
• **Medium adherence (score = 6 - <8)**	53 (12.6)
• **High adherence (score = 8)**	11 (2.6)
	**Mean ± SD**
**8-MMAS score**	3.13 **±** 2.03

The average PAM13 score is 51.93 (standard deviation = 8.54), indicating a low level of confidence and sufficient knowledge to take action. Level 2 scores are between 47.1% and 55.1.

### Quality of life (QOL) by eq. 5D 5 L

The majority of our population had no problems with mobility (55.3%), and only three individuals had extreme problems and were unable to move with a percentage of (0.7%). A significant portion of our population can look after themselves with a percentage (68.4%), and only a very small number of people were unable to do so with a percentage (0.7%). Eight patients were unable to do their usual activities, which represents a percentage of 1.9%. This is compared to the approximately half of the population that was able to perform their typical activities such as working, studying, and performing housework. When it came to pain and discomfort, the vast majority of our population was experiencing either none or only slight problems, with the respective numbers being 173 (41.1%), and 141 (33.5%). In addition, there were only 19 patients who exhibited severe to extreme problems, representing 4.6% of the total population. Table 3.

**Table 3:** Quality of life outcomes by EQ-5D-5 L

Quality of life outcomes	*N* (%)
**Mobility**	
• Level 1 (No problem)	233 (55.3)
• Level 2 (Slight problems)	109 (25.9)
• Level 3 (Moderate problems)	55 (13.1)
• Level 4 (Severe problems)	21 (5)
• Level 5 (Extreme problems/Unable to do)	3 (0.7)
**Self-care**	
• Level 1 (No problem)	288 (68.4)
• Level 2 (Slight problems)	74 (17.6)
• Level 3 (Moderate problems)	44 (10.5)
• Level 4 (Severe problems)	12 (2.9)
• Level 5 (Extreme problems/Unable to do)	3 (0.7)
**Usual activities (e.g. work**,** study**,** housework**,** family or leisure activities)**	
• Level 1 (No problem)	242 (57.5)
• Level 2 (Slight problems)	110 (26.1)
• Level 3 (Moderate problems)	38 (9)
• Level 4 (Severe problems)	23 (5.5)
• Level 5 (Extreme problems/Unable to do)	8 (1.9)
**Pain/Discomfort**	
• Level 1 (No problem)	173 (41.1)
• Level 2 (Slight problems)	141 (33.5)
• Level 3 (Moderate problems)	64 (15.2)
• Level 4 (Severe problems)	31 (7.4)
• Level 5 (Extreme problems/Unable to do)	12 (2.9)
**Depression/Anxiety**	
• Level 1 (No problem)	259 (61.5)
• Level 2 (Slight problems)	97 (23)
• Level 3 (Moderate problems)	46 (10.9)
• Level 4 (Severe problems)	12 (2.9)
• Level 5 (Extreme problems/Unable to do)	7 (1.7)

### Association between medication adherence and patients’ characteristics

In the association between medication adherence and baseline characteristics, the gender was not significant as almost the same percentage of males and females with low medication adherence were the same (almost 85%). We found no correlation between employment and medication adherence but the employed population shows the least number of high medication adherence and that’s reasonable as they have less spare time. At the socioeconomic part, the results were significant in the correlation between economic status and medication adherence although the population that has a salary of fewer than 5000 riyals were having the highest number of population with high medication adherence in addition to the highest number of medium medication adherence it also accomplished the highest percentage of the high and medium medication adherence compared to the low one. Education did not significantly correlate with medication adherence due to that our population is educated. On the other hand, housing status affected medication adherence significantly, as the independent patients that didn’t have anyone to give them their medication had the least medication adherence while patients that were under constant care or at an elderly care home showed higher medium and high medication adherence. Table 4.

**Table 4:** Association between medication adherence and baseline characteristics

Variables		Medication adherence			P. value
		Low	Medium	High	
**Sex**	**Male**	251	37	6	0.533
	**Female**	106	16	5	
**Nationality**	**Saudi**	353	53	11	0.696
	**Non-Saudi**	4	0	0	
**Employment**	**Unemployed**	66	12	3	0.155
	**Employed**	104	7	2	
	**Retired**	187	34	6	
**Socioeconomic status**	**Less than 5000**	96	28	7	**0.003**
	**5000 - <10,000**	92	11	2	
	**10,000 - <15,000**	80	8	0	
	**15,000–20,000**	51	4	2	
	**More than 20,000**	38	2	0	
**Education**	**Primary school**	39	8	3	0.123
	**Secondary school**	42	7	0	
	**High school**	98	17	0	
	**College**	178	21	8	
**Housing status**	**Independent**	318	44	8	**0.047**
	**Under constant care**	35	9	2	
	**Elderly care home**	4	0	1	

**Note: Statistically significant values are presented in bold**

### Association between medication adherence and quality of life

Regarding the association between medication adherence and QOL, no significant result was shown in mobility; however, patients with severe problems and patients unable to move had a low medication adherence level. The same was for self-care as no significant correlation was found in the self-care section. Moreover, patients with moderate problems, severe problems, and extreme problems had no high medical adherence at all. The presence of pain or discomfort had no significant role in medication adherence as the results were not significant. Anxiety and depression also had no significant role in affecting medication adherence as the results had no difference among low, medium, or high. Table 5.

**Table 5:** Correlation between medication adherence and quality of life

Variables		Medication adherence			P. value
		Low	Medium	High	
**Mobility**	**Level 1 (No problem)**	193	33	7	0.902
	**Level 2 (Slight problems)**	94	12	3	
	**Level 3 (Moderate problems)**	47	7	1	
	**Level 4 (Severe problems)**	20	1	0	
	**Level 5 (Extreme problems/Unable to do)**	3	0	0	
**Self-care**	**Level 1 (No problem)**	235	43	10	0.368
	**Level 2 (Slight problems)**	66	7	1	
	**Level 3 (Moderate problems)**	42	2	0	
	**Level 4 (Severe problems)**	11	1	0	
	**Level 5 (Extreme problems/Unable to do)**	3	0	0	
**Usual activities**	**Level 1 (No problem)**	203	34	5	0.539
	**Level 2 (Slight problems)**	95	12	3	
	**Level 3 (Moderate problems)**	31	6	1	
	**Level 4 (Severe problems)**	21	1	1	
	**Level 5 (Extreme problems/Unable to do)**	7	0	1	
**Pain/Discomfort**	**Level 1 (No problem)**	145	24	4	0.271
	**Level 2 (Slight problems)**	120	16	5	
	**Level 3 (Moderate problems)**	51	12	1	
	**Level 4 (Severe problems)**	30	1	0	
	**Level 5 (Extreme problems/Unable to do)**	11	0	1	
**Anxiety/Depression**	**Level 1 (No problem)**	218	35	5	0.109
	**Level 2 (Slight problems)**	83	10	4	
	**Level 3 (Moderate problems)**	41	5	0	
	**Level 4 (Severe problems)**	9	1	2	

## Discussion

We conducted a cross-sectional study to explore the influence of population characteristics and health image on medication adherence in the elderly population in Saudi Arabia. Our goal was to find out if medication adherence is essential for improving clinical outcomes and health in the elderly.

Medication adherence is well known to be influenced by several external factors, including the patient’s psychological status as well as the patient’s community and culture [27]. Overtreatment of a disease is possible if patients do not take their prescribed medications as directed, which increases the risk of side effects, and hospitalizations [28]. This is besides the increased costs of handling the problems and associated conditions [29].

Most of our elderly population was nonadherent. Nonadherence was attributed in our study to forgetfulness, difficulties in managing medication, concerns about side effects, doubt about the need for the medication, and a lack of trust in some medicines, demonstrating that the elderly’s behaviors, beliefs, and attitudes influence medication adherence [30]. Other times they just stop taking medication as they feel that the medicine they take makes them even worse, or at another time they feel better so they stop the medication on their own. Most of our population feel hassled about sticking to their treatment plan. All these results led to the MMAS class being lower than the six-point score; 84.8% at low adherence. On the other hand, a previous study in 146 elderly patients in Norway found that moderate or high adherence (MMAS-8 score ≥ 6) was demonstrated by 83% of the patients and that there was no association of medication complexity, age, or other variables with medication adherence [31]. Nevertheless, A study in the United States conducted on 1391 elderly patients found that only 9.56% were low-adherent to the medication [13]. We summarized the medication adherence rates among elderly patients with chronic diseases in different countries, based on the MMAS-8 score in Table 6. Our study has the lowest rate of moderate or high adherence (15.2%) and the highest rate of low adherence (84.8%) among the studies included. This indicates that medication adherence is a serious problem in Saudi Arabia and that there is a need for more interventions and strategies to improve it. Our study also contributes to the scientific literature by providing a comprehensive assessment of the factors associated with medication adherence, such as socio-demographic characteristics, health status, medication-related factors, and QOL.

**Table 6:** Prevalence of Medication adherence in elderly people around the world

Study ID	Country	Number	Age (yrs.)	Moderate or high adherence (MMAS-8 score ≥ 6), (%)	Low adherence (score = < 6), (%)
**Our study**	Saudi Arabia	421	60.4 ± 8.1	64 (15.2%)	357 (84.8%)
**Parker et al. 2019** [31]	Norway	157	76 ± 7.2	130 (83%)	27 (17%)
**Muntner et al. 2013** [13]	United States	1391	75.1 ± 5.5	1258 (90.4%)	133 (9.6%)
**Holt et al. 2013** [12]	United States	1075	≥ 75	948 (88.2%)	147 (11.8%)
**Berni et al. 2011** [14]	Italy	42	70 ± 10	25 (60%)	17 (40%)
**Lee et al. 2013** [40]	China	1114	65.7 ± 11.1	725 (65.1%)	389 (34.9%)
**Okello et al. 2016** [15]	Uganda	121	> 60	22 (18.2%)	99 (81.8%)
**Jankowska-Polańska et al. 2017** [41]	Poland	180	≥ 66	146 (81.1%)	34 (18.9%)
**He et al. 2023** [42]	China	436	72.5	224 (51.4%)	212 (48.6%)
**Khayyat et al. 2017** [17]	Saudi Arabia	49	> 65	28 (57%)	21 (43%)
**Al-Rahmahi et al. 2015** [16]	Palestine	150	≥ 65	72 (48%)	78 (52%)
**Allahem et al. 2022** [43]	United Arab Emirates	117	≥ 66	97 (82.91%)	20 (17.09%)

Our study showed a non-significant correlation between medication adherence with sex, nationality, employment, and education. Although the ability to read and comprehend medication instructions is one factor that can affect patient compliance all of our population can read, as they had at least a primary education grade [32]. Moreover, according to a previous study, gender, personality, and cultural factors may also influence adherence [33], Unlike gender, which had no effect, we found a significant relationship between the socioeconomic status and the housing situation of the people. However, we did not examine the cultural factors because all the participants belonged to the same Arabian culture [34]. The economic status may inflict a human nature of fearing comorbidities that the population with low income cannot afford if they stopped taking the medication and showed low medication adherence. On the other hand, those with higher income would feel more secure that they can deal with the complications that will result due to the irregularity in taking their medication. That may be the psychological reason for the population with a higher salary to show lower medication adherence than the population with a lower salary. At housing point, it has been insured in many previous studies that elder living with their family, or at a care home have higher medication adherence and medical attention than others who live alone [34–36].

We found that the average PAM13 score is 51.93 (standard deviation = 8.54), indicating a low level of confidence and sufficient knowledge to take action. Level 2 scores are between 47.1% and 55.1. A previous study in Hungary on 733 elderly people found that the Mean (± SD) PAM-13 score was 60.6 ± 10. Another paper conducted by Overbeek et al. in the Netherlands established that the median PAM score was 51 [37].

Regarding the mobility outcome by EQ-5D-5 L score, The majority of our population had no problems with mobility (55.3%), and these results were similar to a previous study in France (43.8%) that had no problems with mobility [38]. On the other hand, Kaambwa et al. conducted a study in Australia and revealed that only (23%) of the population had no problems with mobility [39].

One of our strengths that all our population can read as the lowest grade in education was a primary grade and it’s known that education influences medication adherence, so we did not include the undereducated population to ensure the results were not affected by education. We are the first cross-sectional study to use 8-MMAS with PAM-13 and EQ-5D-5 L in Elderly people in Saudi Arabia. Also, the sample size was calculated and achieved, and we have done most of the available analyses investigating different relationships. However, our study had some limitations; as our study is based on self-reporting which is not the most accurate way to get data, especially with elders as they are more susceptible to bias due to their memory in recalling or reporting. In addition, our population did not share a common disease, they only share that their disease is chronic. Also, we did not find validation in the Arabic version of the PAM 13 tool, so we did a pilot sample on our population by using a translator and we found the results were reliable to use in the rest of our population.

Future research should explore more comparison points with larger populations with a focus on the cost of the chronic medication that the population has to stick to and explore the number of medications used by each patient so the comparison among the population can be fairer.

## Conclusion

Medication adherence was generally low in the old Saudi Arabian population. Housing status has a direct effect on medication adherence as patients who have someone to remind them of medication time have higher medication adherence than independent patients living alone. Socioeconomics also has a significant effect on the patient’s medication adherence, possibly due to psychological effects and the feeling of the lower-salary population that if their condition became complicated, they will have to afford the extra money to be cured of these co-morbidities. Therefore, awareness of the necessity of adherence to medication for the elderly is essential. Finally, we did not find any correlation between medication adherence and quality of life.

## Data Availability

Data are available upon reasonable request. Data are available from the corresponding author upon reasonable request.
